# Monthly Summary

**Published:** 1894-08

**Authors:** 


### Monthly Summary.

ELECTRICITY IN DENTISTRY.—By the use of an electric light in connection with the little mirror introduced into the mouth the teeth and alveolar processes are brilliantly illuminated and rendered translucent. Thus anything wrong about the teeth may be quickly discovered. Perhaps the dead tooth may be hidden in the jaw, never having been erupted, and may have been the obscure cause of trouble for years. The light reveals it at once. Facial neuralgia, by the way, is nearly always due to a dead tooth. Electricity is most valuable as a motive power for tooth boring tools, which, strange to say cause less pain the faster they go. Most people now grown up can recall the excruciating pain caused by the excavating instrument which the dentist of a generation ago slowly revolved between his fingers. The "burs" now made for such work are much finer than they were half a dozen years ago, being capable of cutting through steel bars. Furthermore, the laborious method of turning them out by hand has been superseded recently by a machine which produces them at a cost of 19 cents apiece.

Electricity is employed also for pulling teeth. To the battery is attached three wires. Two of them have handles at the end, while the third is attached to the forceps. The patient grasps the handles, the electricity is turned on suddenly, and the dentist simultaneously applies his forceps to the tooth. The instant the tooth is touched, it, as well as the surrounding parts, becomes insensible to pain. A jerk, and it is out.—*Providence Journal.*

---

THE DANGERS OF HYPNOTISM —Prof. Grainger Stewart, of Edinburgh University, in discussing the merits of hypnotism as a therapeutic agent, lays down the following propositions (*Hosp. Gaz.*): 1, That hypnotic conditions of all varieties are practically a diseased state of the nervous system, an artificial neurosis, 2, that hypnotism is undoubtedly able to modify certain morbid processes of the nervous system, particularly those of a functional kind; 3, that in certain conditions results undoubtedly favorable, at least for the time, had been obtained by hypnotic treatment; but, 4, that in every case hypnotic treatment involves hazard to the nervous system that those who are the most susceptible to its influence are the most apt to suffer, and that though it might free the patient from one set of symptoms, it is apt to make him the victim of many others. He considers that hypnotism should be employed very rarely to the very moderate extent, and only after careful study of the patient's condition. We would even go farther than Professor Stewart, and say that no one should employ hypnotism as a therapeutic agent under any circumstances.—*Canada Lancet.*

---

THE NAILS.—Sulphur is especially useful in improving the nutrition of the nails. Sulphur is a normal ingredient of nail-tissue, in which it exists in a comparatively large proportion. It is consequently an excellent remedy in cases where the nutrition of the nails is perverted. In such conditions sulphur may be justly regarded as a specific nutrient remedy.

The mode of administration is an important point. It should be given regularly for a lengthened period in minute doses, such as five grains three times a day. Administered in this manner sulphur not only directly supplies the nail with an element necessary to its healthy life, but it also exerts a beneficial influence upon the composition of the blood. Sulphur is of material assistance in the treatment of the constitutional disorders upon which the trophic changes in the nails depend. This remedy possesses a decided value in the management of chronic rheumatism. From its action upon the liver and intestinal glands, sulphur is noticable in the treatment of anemia and chlorosis. I have found sulphur of much value when the nails are brittle and marked by white spots or ridges. As an excellent local application in the same conditions, I can recommend an ointment containing from ten to sixty grains of the oleat of tin to the ounce of excipient To this, for the sake of elegance may be added a little carmine. When rubbed along the nail and the surface surrounding it, the ointment of tin oleate improves the structure and luster of the nail—*Medical and Surgical Reporter.*

---

TO SET A LOGAN CROWN.—The following method of setting a Logan crown, which is by no means new, will in the majority of cases enable a perfectly close joint to be made between the crown and root-end. Cut several small pieces, about one-quarter inch square, from a strip of thin articulating paper. In the center of each punch a hole with the coffer-dam punch, about the size of the largest hole made by the Ainsworth punch. Having prepared the root-end, slip the perforated piece of articulating paper over the pin of the Logan crown and press it firmly into position, in contact with the root. On withdrawing the crown and removing the articulating paper, the points of contact will be found to be marked black. Grind these off carefully, readjust on the root as before, grind again, and continue the operation of fitting and grinding till the mark made by the articulating paper on the contact surface of the crown presents as a uniformly unbroken black ring. When this has been accomplished, the crown will be found to fit the root-end with the utmost accuracy. The advantages of fitting a crown directly to the root are, it would seem, self-evident from the mechanical standpoint, and involve besides the least expenditure of time.—*Cosmos.*

---

DIAGNOSIS OF PULP STONES.—Persistent resistance to arsenic is a good diagnostic symptom of pulp stones. "Persistent pain that you cannot stop means pulp stones, and it means it always. There is no question about it."—*Ottolengui.*

SILVER AND ALUMINUM.—The utilization of the well-known property of aluminum to lower the fusing point of iron is a very neat and clever application of a curious phenomenon.

Silver is the metal which seems most useful in improving alumin um. Five per cent silver gives to aluminum elasticity, which is wanting in the pure metal, increases its hardness, and is capable of being polished, and does not injure its malleability, or much increase the weight. This alloy is excellent for handpieces and handles of instruments. It is about as hard as coin silver, and is not affected by sulphuretted hydrogen; hence it does not easily tarnish.—*British Journal.*

---

HINTS WORTH REMEMBERING.—To remove the stains of tincture of iodin from either the hands or napkins, apply strong ammonia. The spots will immediately come out clear.

The stains of nitrate of silver, on either the hands or napkins, can be easily removed. First, cover the spots with tincture of iodin, wait a few moments, then apply strong ammonia, and rub well. If on the hands and not discovered till the hands are washed, black stains will be well set, but proceed as above and they will disappear at once.—*International.*

---

PROTECTION OF CEMENT FILLINGS.—Instead of parrafin which scales off as soon as wet, melt together rosin and wax on a spatula, and pour on the filling after it has stood a few minutes. After a day or two they will take a polish almost like ivory.—*Items of Interest.*

CORRECT ARTICULATION IN BRIDGE-WORK.— Place on the gum, between the pier teeth, a bolster of wax, and put in position the pier crown and the dummies as they have been cemented together with wax on the articulating model. Have the patient bite on them, and when each tooth is in proper position, invert on the jaw a partial tray filled with a half-and-half mixture of marble dust and plaster, mixed with water primed with sulphate of potash. Remove from the mouth carefully, clean off the wax, and the piece is invested, ready for soldering.—*Wm. Crenshaw in the Items of Interest.*

---

COCAIN INJECTION.—To eliminate all danger in the injection of cocain, Gauthier gives the following formula:

R. Cocain hydrochlo - - - - grs. iij.
Alcoholic solution nitro glycerin 1 per cent. M. x.
Distilled water. - - - - - fl. dr. iiss.

Each syringeful, (16 M.) contains ⅓ gr. cocain and 1 M. nitro-glycerin solution. Instead of collapse, the face flushes, and the heart beats quicker and stronger, and the patient is altogether comfortable.—*Am. Med. and Surg. Bulletin.*

---

ALLOY FOR GOLD SOLDERS.—Equal parts by weight of copper, silver and zinc. Melt copper and silver in a crucible, and gradually add the zinc in small pieces. When the blue flame of gas is thrown off, pour into an ingot and label "alloy for gold solder." Take of this alloy one part, and of the gold plate you are using, three parts, and you will have a solder that will melt easily, flow smoothly, and will not change color in the mouth.—*Items of Interest.*

